# Complete genome sequences of *Lactococcus petauri* strains 473AN and 473GN isolated from the blood culture of a Japanese patient with infective endocarditis

**DOI:** 10.1128/mra.00437-24

**Published:** 2024-06-28

**Authors:** Masayuki Imajoh, Marina Suzuki, Akira Okamoto, Maiga Salif, Riku Ogawa, Aguri Mikami, Masanori Daibata

**Affiliations:** 1 Faculty of Agriculture and Marine Science, Graduate School of Integrated Arts and Science, Kochi University, Kochi, Japan; 2 Department of Bioresource Production Science, The United Graduate School of Agricultural Sciences, Ehime University, Ehime, Japan; 3 Department of Clinical Laboratory, Tokyo Metropolitan Toshima Hospital, Tokyo, Japan; 4 Department of School Health Sciences, Aichi University of Education, Aichi, Japan; 5 Department of Microbiology and Infection, Kochi Medical School, Kochi University, Kochi, Japan; Wellesley College, Wellesley, Massachusetts, USA

**Keywords:** *Lactococcus petauri*, infective endocarditis, blood culture, human

## Abstract

Here, we report the complete genome sequences of *Lactococcus petauri* strains 473AN and 473GN, isolated from the blood culture of a Japanese patient with infective endocarditis. The complete genomes of 473AN and 473GN consist of single chromosomes of 2,065,772 and 2,094,461 bp, respectively.

## ANNOUNCEMENT

Infective endocarditis (IE) results from bacterial infection of native or prosthetic cardiac valves, the endocardial surface, or indwelling cardiac devices (1). In Japan, the annual incidence of IE is 32.4 cases per 1,000,000 individuals, with *Staphylococcus aureus* being the most primary causative agent (2). *Lactococcus garvieae* is an unusual causative agent of IE, which was first reported in 2011 in Japan (3). Blood culture plays a pivotal role in diagnosing IE, as a positive result is indicative of the condition (4). Strains 473AN and 473GN were isolated on sheep blood agar medium by incubation under 5% CO_2_ gas at 35℃ overnight from the blood culture of an octogenarian Japanese female patient with IE who was traditionally diagnosed with Duke’s criteria (5), and identified as *L. garvieae* using the Rapid ID32 Strep API system (SYSMEX bioMérieux, France). Informed consent was obtained from the patient and her family according to the Declaration of Helsinki. Herein, we report the complete genome sequences of 473AN and 473GN.

Frozen stocks of 473AN and 473GN were cultured on brain heart infusion (BHI) agar plates (BD Difco, USA) at 37°C overnight. Single colonies were incubated with shaking in 10 mL of BHI broth at 37°C overnight. Following centrifugation at 16,000× *g* for 2 min, genomic DNA was extracted from bacterial pellets using the Wizard HMW DNA Extraction Kit (Promega, USA) according to the manufacturer’s instructions and used for Illumina and Nanopore sequencing. For Illumina sequencing, genomic DNA was fragmented and processed to achieve an average size of 330 bp using the QIAseq FX DNA Library Kit (QIAGEN, USA). After adaptor ligation, 20 pM pooled libraries were sequenced on an Illumina MiSeq (MiSeq Reagent Kit v.3; 150 cycles, USA), generating 75 bp paired-end reads. Miseq raw reads with Phred-encoded quality scores above 30 were selected for assembly using Trim Galore v.0.6.5 (6). For Nanopore sequencing, genomic DNA was treated with the 1D Ligation Sequencing Kit (SQK-LSK109, Oxford Nanopore Technologies, USA) with Native Barcoding Expansion (EXP-NBD104). Following adaptor ligation, the pooled libraries were sequenced on a MinION with an R9.4.1 flow cell using MinKNOW software v22.05.5. Base calling was performed using Guppy v.4.2.3 in the accurate mode implemented in MinION software. MinION raw reads below a minimum quality score of 8 were discarded using NanoFilt v.2.8.0 (7). Unicycler v.0.4.8 (8) was used for hybrid assembly of Miseq and MinION reads, with its default parameters applied. Genome error correction, circularization, and rotation were implemented in the Unicycler pipeline. Gene annotation, taxonomy verification, and genomic average nucleotide identity calculations were performed using the DDBJ Fast Annotation and Submission Tool v. 1.2.0 (9).

The complete genome characteristics of 473AN and 473GN are presented in Table 1. Both strains belonged to *Lactococcus petauri*, a species newly described in 2017 (10). Routine methods, including use of the Rapid ID32 Strep API system, MALDI-TOF MS, and PCR assays, cannot differentiate between *L. garvieae* and *L. petauri* (11). Consequently, *L. petauri* may have been erroneously classified as *L. garvieae* and potentially constitutes a causative microorganism regarding IE.

**TABLE 1:** Characteristics of the complete genome sequences of *L. petauri* 473AN and 473GN

	473AN	473GN
Number of contigs	1	1
CheckM completeness (%)	99.02	98.6
Structure	Circular chromosome	Circular chromosome
Number of Miseq reads	4,022,826	2,466,248
Number of MinION reads [N50 value (bp)]	25,736 (17,088)	13,089 (15,648)
Chromosome size (bp)	2,065,772	2,094,461
guanine-cytosine (GC) content (%)	38.2	38.2
Coverage (×)	418	237
N50 value (bp)	2,065,772	2,094,461
Number of coding sequences	2,083	2,135
Number of rRNAs	16	16
Number of tRNAs	62	62
Whole-genome sequence accession no.	AP031392	AP031393
DRA accession no. of Illumina MiSeq and MinION data	DRX528550 and DRX528551	DRX528552 and DRX528553
gANI values of strains in *L*. *petauri* and *L*. *garvieae*		
*L*. *petauri* 159469 (GCA_002154895.1)	97.7782	97.8802
*L*. *garvieae* DSM 20684 (GCA_002441675.1)	93.3425	93.4427
*L*. *garvieae* FDAARGOS_929 (GCA_016026695.1)	93.3143	93.4303
*L*. *garvieae* NBRC100934 (GCA_000739975.1)	93.2732	93.3445
*L*. *garvieae* subsp. *garvieae* CCUG 32,208T (GCA_008692985.1)	93.2138	93.3048

## Data Availability

The complete genome sequences of *L. petauri* 473AN and 473GN are available at National Center for Biotechnology Information under accession numbers AP031392 and AP031393, respectively. Moreover, raw sequence data from Illumina MiSeq and MinION are available at DDBJ Sequence Read Archive under DRA accession numbers DRX528550 and DRX528551 for 473AN and DRX528552 and DRX528553 for 473GN.
